# Weather patterns and occurrence of epileptic seizures

**DOI:** 10.1186/s12883-021-02535-8

**Published:** 2022-01-21

**Authors:** Sanja Tomasović, Josip Sremec, Jelena Košćak Lukač, Gordana Sičaja, Koraljka Bačić Baronica, Vedran Ostojić, Zurap Raifi, Nada Tomić Sremec, Dunja Plačko-Vršnak, Lidija Srnec, Krunoslav Mikec

**Affiliations:** 1 Department of Neurology, “Sveti Duh” University Hospital, Sveti Duh 64, 10000 Zagreb, Croatia; 2 Josip Juraj Strossmayer Medical School, University of Osijek, Josipa Huttlera 4, Osijek, Croatia; 3 Catholic University of Croatia, Ilica 242, Zagreb, Croatia; 4 Department of Laboratory Diagnostics, University Hospital Center Zagreb, Kišpatićeva 12, Zagreb, Croatia; 5 Croatian Meteorological and Hydrological Service, Ravnice 48, Zagreb, Croatia

**Keywords:** Epilepsy, Seizure, Meteorology, Weather, Biometeorology

## Abstract

**Background:**

The results of various studies that have so far evaluated the influence of meteorological conditions on seizures are often divergent. No studies have been performed so far that evaluate the possible correlation between the occurrence of seizure-related events, surface and upper level atmospheric conditions and biometeorological forecasts. The aim of this study is to investigate those interactions.

**Methods:**

Records of “Sveti Duh” University hospital, Zagreb, Croatia between January 2016 and May 2020 were analysed in order to determine number of patients per day who were assessed through Emergency room because of a recent epileptic seizure. The dates were assessed for surface and upper level atmospheric conditions and biometeorological forecasts. Analyses of these factors were performed for two consecutive days preceding that day, the day of seizure, as well as for the following day. Data was analysed using chi-squared test, Mann-Whitney U-test or Kruskal-Wallis test (with Conover’s test for post-hoc analyses), depending on variable type. Additionaly, logistic regression was used to study effects of variables on seizure occurrence. Statistical significance was set to *p* < 0.05.

**Results:**

Results showed significant difference between the numbers of patients with seizure depending on upper level type on the following day. We also observed fewer daily patients with seizures when the synoptic situation on the following day was high pressure field then when it was low pressure or non-gradient pressure field (observed just during the colder part of the year), which was supported in our regression analyses. A greater frequency of seizures was observed if the biometerological prognosis was deemed unfavourable on the following day. Furthermore, our results showed significantly larger proportion of days with seizure patients in warmer, than in colder days of the year.

**Conclusions:**

All of the analyzed weather-related parameters seem to be associated with daily numbers of seizures on the previous day. The reason behind this phenomenon is uncertain; however, the results indicate that weather patterns have a certain influence on patients with epilepsy, but are not yet fully understood or adequately appreciated.

## Background

Various environmental conditions have been studied extensively for their role as precipitating or modifying factors related to human health. Particularly, meteorological and climate factors, both short and long-term, have been a subject of interest for health professionals for years, without unequivocal consensus about their role, be it a direct [1–4], or an indirect role, such as a predisposition for the propagation of infectious diseases [5–7].

In neurology, surface meteorological conditions such as mean daily temperature, humidity, atmospheric pressure and wind conditions, as well as seasonal changes, have been associated with the occurrence of disorders with paroxysmal onset: seizure related disorders, as well as diseases of vascular origin (haemorrhagic and ischemic stroke, subarachnoid haemorrhage), although the results of various studies for the latter are often heterogeneous and conflicting [8–12].

When it comes to epilepsy and seizure related disorders, studies that have so far been carried out often have conflicting and inconclusive results. A negative correlation was found between mean air temperature and paediatric seizures [13]. In another study, occurrence of febrile seizures in children was not associated with weather conditions, but was correlated to epidemic febrile infections [14]. Seizure frequency in patients with epilepsy was observed to be higher in correlation with a significant change in atmospheric pressure in one study [15], but not in a later study by the same lead author [16]. In another study, a negative correlation was found between seizure occurrence and absolute atmospheric pressure values [17]. The same study noted a correlation between seizure occurrence and high humidity and lower mean air temperatures. A significant association was found between seizure frequency, higher humidity, and lower ambient temperatures, but contrary to the previously mentioned study, also a significant relation between higher atmospheric pressure and seizure incidence. Furthermore, a higher seizure occurrence in the winter days and days with lower daylight duration was noted [18]. Study by Payne et al. showed that current temperature, wind speed, humidity and rainfall performed better than chance in predicting upcoming seizure in at least one patient. On the contrary, pressure, maximum and minimum temperature did not contribute to seizure likelihood. However, weather features showed the worst forecasting performance among those analysed [19]. Results of another study suggest that temperature is the only meteorological factor among observed which affects seizure occurrence. Humidity, atmospheric pressure, precipitation, and number of hours of sunshine were not correlated to seizure incidence [20]. Chiang et al. also showed that ambient temperature affects seizure occurrence. Moreover, they noted that some air pollutants, including CH_4_ and NO, are positively correlated with seizure incidence [21]. Not only air pollution, but climate change, including frequent extreme weather events may trigger seizures [22]. Other studies show no correlation between seizures and the aforementioned weather conditions, but establish a tendency for an asymmetric circadian distribution of seizures [23]. Unstable weather conditions correlate with epileptiform changes in electroencephalography (EEG) studies of patients with epilepsy [24].

As per our current knowledge, no studies that evaluate the possible correlation between the occurrence of seizure-related events and ground and upper level atmospheric conditions, biometeorological forecasts, and temperature at various altitudes have been conducted so far. Our aim is to investigate those possible interactions.

## Methods

Emergency room records of “Sveti Duh” University Hospital, Zagreb, Croatia between 1 January 2016 and 20 May 2020 (1600 days) were analyzed in order to determine the number of patients that have been assessed through the emergency room on each individual date because of a recent epileptic seizure. The patients complied with inclusion criteria if the ICD-10 codes assigned to them during the assessment pertained to epilepsy or seizure related disorders. Patient histories of those patients were afterwards analyzed to determine if a recent seizure was indeed the reason for their visit to the emergency room, and to attempt to eliminate other disorders that could mimic seizure disorders (psychogenic non-epileptic seizures (PNES), transient loss of consciousness of other origin, certain psychiatric disorders). All types of focal or generalized epileptic seizures were eligible, with no discrimination regarding current event or prior epilepsy severity. Patient anonymity was sustained throughout the study as data that could identify a patient were not available to any of the researchers (diagnoses, dates of visit, and patient histories are assigned to single common identifying codes in the hospital informatics system, and are retrieved without the need for further patient identification). The study was approved by the hospital ethics committee.

Meteorological data were supplied and analysed by the Croatian Meteorological and Hydrological Service. Upper level weather patterns, surface synoptic situations and biometeorological forecasts were determined for each individual date, and associations between the daily amount of patients with seizures and aforementioned meteorological factors were determined. Afterwards, analyses of same meteorological factors were performed for two consecutive days preceding that day, as well as for the next day. This enabled us to additionally determine if there is a relationship between meteorological situations that preceded the seizure, as well as to determine if seizures have a relationship with weather patterns and conditions in near future.

The analysis of the upper level of the atmosphere was carried out by 500 hPa geopotential field analysis charts (located approximately at 5500 m above the surface) at 00 UTC (Coordinated Universal Time). These isobaric charts represent constant pressure surfaces, i.e. different altitude levels for 500 hPa pressure. The contours effectively show the main tropospheric waves that “control” our weather - low heights indicate troughs and cyclones in the middle troposphere whilst high heights indicate ridges and anticyclones. This pressure level is often termed the “steering level”, as it is thought to have a significant influence on forming the weather systems beneath it (near to the Earth’s surface), which roughly move in the same direction as the winds at the 500 hPa level. The top of that part of the atmosphere in which our weather is formed is known as the tropopause and is at about 11,000 m, so the isobaric surface of 500 hPa most often represents the majority of the flow in the troposphere (from surface to tropopause) [25].

Upper level weather regimes over Croatia have previously been classified differently by different authors [26–28]. In our study the upper level weather types were separated into the following categories, depicted in Table 1, [29] in relation to local movements and the distribution of atmospheric pressure fields and gradients.

**Table 1:** Upper level weather types - description and abbreviations

Ridge (R)	A ridge is an elongated area of relatively high pressure extending from the centre of a high-pressure region. The upper level ridge (the height contours bend on upper level synoptic chart strongly to the north) are accompanied usually by warm and dry weather conditions at the surface.
Non gradient field in ridge (NG-R)	There is no significant flow of air in the upper levels of the atmosphere. For a more detailed analysis we could discriminate non-gradient field in ridge (NG-R) and in trough (NG-T). The weather at the surface can be stable, without any wind, but also there can be local instability as well due to convective development, especially in summer.
Non gradient field in trough (NG-T)	There is no significant flow of air in the upper levels of the atmosphere. For a more detailed analysis we could discriminate non-gradient field in ridge (NG-R) and in trough (NG-T). The weather at the surface can be stable, without any wind, but also there can be local instability as well due to convective development, especially in summer.
Front side of the ridge (GNW)	Transitional state between ridge and trough where flow in upper level move from northwest to southeast. The north-westerly flow at the front side of the ridge tends to bring colder and sometimes more humid air.
Back side of the trough (NWS)	Transitional state between ridge and trough where flow in upper level move from northwest to southeast, bringing usually colder and drier air. At surface weather could be associated with stable and windy weather.
Back side of the ridge (GSW)	Transitional state between trough and ridge where flow in upper level move from southwest to northeast, bringing usually warmer and wetter air. At surface weather could be associated with changeable clouds and moderate to strong wind.
Front side of the trough (SWS)	Transitional state between trough and ridge where flow in upper level move from southwest to northeast, bringing usually warmer and wetter air. At surface weather could be associated with changeable clouds, sometimes with occasionally rain, moderate to strong wind and warmer conditions than before.
Trough (T)	A trough is an elongated area of relatively low pressure extending from the center of a region of low pressure. The upper level troughs (the height contours bend on upper level synoptic chart strongly to the south) are typically preceded by stormy weather and colder air at the surface.
Upper level low (ULC)	Upper level lows are closed cyclonically circulating eddies in the middle and upper troposphere. They are sometimes also called “cold drops”, because the air within an Upper level low is colder than in its surroundings. Upper level lows have been responsible for bringing high amount of precipitation (e.g. heavy snow in the winter) especially if it is stationary. It may or may not have to be connected to surface low.
Northerly flow (NS)	Air in upper level move from north to south, with parallel height contours on upper level synoptic chart, bringing usually colder air. The weather at the surface could be sunny but chilly.
Northeasterly flow (NES)	Air in upper level move from northeast to southwest, with parallel height contours on upper level synoptic chart. The weather at the surface could be accompanied by colder and windy conditions.
Southerly flow (SS)	Air in upper level move from south to north, with parallel height contours on upper level synoptic chart. At surface weather could be associated with strong and stormy wind, sometimes accompanied by precipitation.
Southeasterly flow (SES)	Air in upper level move from southeast to northwest, with parallel height contours on upper level synoptic chart. The weather at the surface could be mild and humid, especially in w inter.
Westerly flow (WS)	Zonal flow, where air in upper level usually move from west to east, with parallel height contours on upper level synoptic chart, tends to result in mild, changeable weather at the surface.
Easterly flow (ES)	Air in upper level move from east to west, with parallel height contours on upper level synoptic chart. The weather at the surface is characterized by strong winds, cold and cloudy weather and very often with precipitation.

**Fig. 1:**
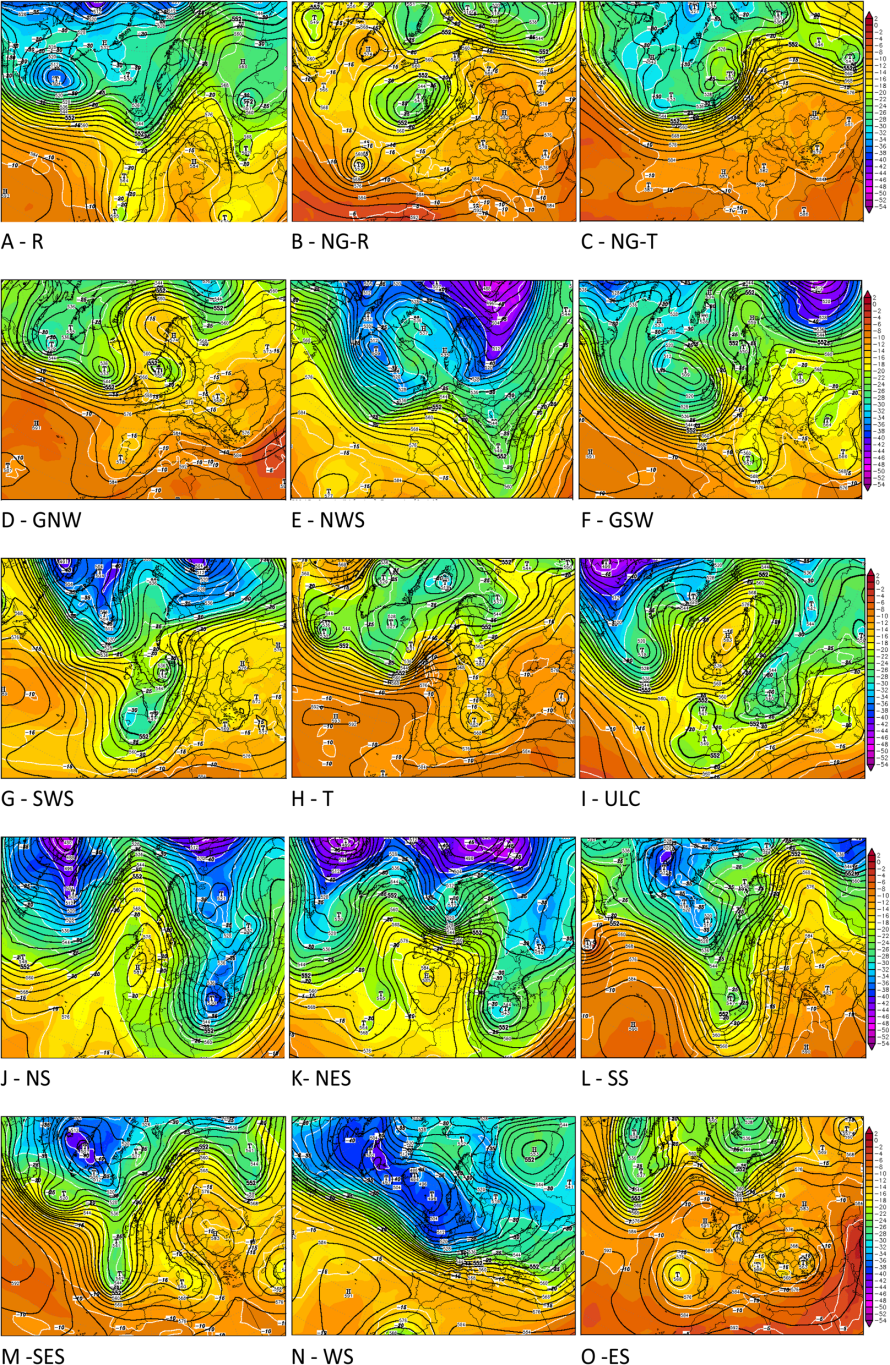
**A**-**O** Examples of upper level weather types from the Table 1 are represented by charts of geopotential height (gpdam) at 500 hPa (black lines) and temperature (°C) at 500 hPa (color shading – 2 degrees interval – values to the right side of the synoptic chart; white lines – isotherms, 5 degrees interval) – source US GFS model (Global Forecast System) - http://www1.wetter3.de/index

Examples of synoptic charts at 500 hPa geopotential height and temperature representing each upper level weather type are provided in Fig. 1.

The synoptic situations were analysed (using DWD (Deutsche Wetterdienst) surface charts at 00 UTC, in some cases with the help of a 850 hPa geopotential field) and classified according to weather types. According to Poje’s classification [30–32], there are 29 weather types, divided into four basic groups of atmospheric systems:

1. Low pressure field (cyclone (5 types), trough (7 types))
2. High pressure field (anticyclone (5 types), ridge, high pressure bridge)
3. Non-gradient pressure field (cyclonic and anticyclonic)
4. Transition state with advection from one of eight flow directions.

A biometeorological forecast is issued twice a week by the Croatian Meteorological and Hydrological Service on their website and through the media. It is a product that is based on current and forecasted weather situations as well as the results of interdisciplinary studies between physicians and meteorologists. It has been shown that certain weather conditions are unfavourable for particular groups of people. Certain weather phases are linked to their influence on chronic patients or patients prone to meteoropathy [33–36], on the basis of which physicians can issue advice on how to adapt. The biometeorological forecast is determined in practice by considering the current synoptic situation and the future weather development. All synoptic situations, either surface or upper-level, can be related to the weather types – similar to the weather patterns used as first meteorological data set described above. According to the Kügler [34], each determined synoptic situation is associated with weather phase (e.g. anticyclonic, cyclonic, prefrontal, frontal, zonal). It means that it gives information about the change of weather or parameters in the atmosphere. Some typical weather situations that can be noticed in Croatia are bora (strong cold northeast wind that brings fresh air, especially in the Northern Adriatic – usually it presents better/relatively favourable situation) and jugo (southern wind that brings unusually warm and humid air and can be very unfavourable). Weather phases are than referred as favourable, relatively favourable or unfavourable and the knowledge on how they influence meteoropatic population or chronical disease is used in the issuing the biometeorological forecast.

Upper level parameters (such as temperature, wind, humidity, etc.), that define weather types influence those same parameters at the surface, and create conditions in the atmosphere that form complete biometeorological situations. All of those can influence human health and potentially have a role in provoking epileptic seizures. The subject of this study is to analyse the possible association between the daily number of patients that have sought medical aid because of an epileptic seizure and all aforementioned meteorological conditions.

Data was analysed using Statistica, version 13.3.0 (TIBCO Software Inc., Palo Alto, CA, USA). Differences between variables were determined using chi-squared test, Mann-Whitney U-test or Kruskal-Wallis test (with Conover’s test for post-hoc analyses), depending on variable type. Logistic regression was used to study effects of variables on seizure occurrence. Statistical significance was set to *p* < 0.05.

## Results

**Fig. 2:**
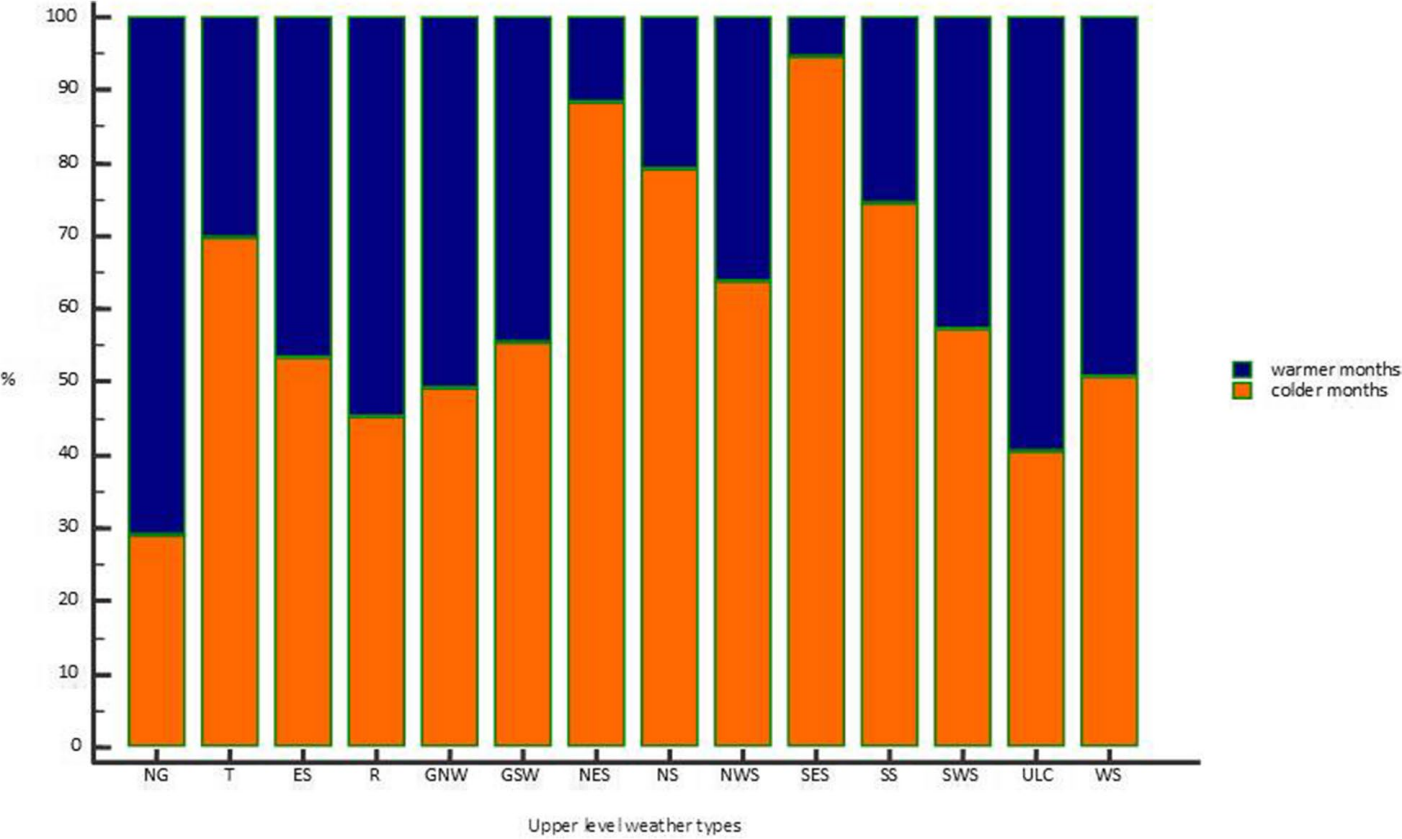
Distribution of upper level weather types in warmer (May through Oct) and colder (Nov through Apr) parts of the year

The distribution of upper level weather types varied significantly throughout the year, both on a monthly basis (*p* < 0.0001, χ^2^ test), and when months are grouped into warmer (May through October) and colder (November through April) categories (*p* < 0.0001, χ^2^ test, Fig. 2). The differences stem from distributions of days with non gradient upper level weather types (for a more detailed analysis we could discriminate non gradient field in ridge (NG-R) and in trough (NG-T) – as descripted in Table 1), which are more common in warmer months, while trough (T), as well as SWS, NWS, SES, NES, NS, and SS types are more common in colder months.

There were no differences regarding the number of patients with seizures between days with particular upper level weather types (*p* = 0.433, Kruskall-Wallis test), as well as in respect to those weather types on the day (*p* = 0.577, Kruskall-Wallis test) or 2 days (*p* = 0.982, Kruskall-Wallis test) earlier. However, there was a significant difference in upper level weather types on the following day (*p* = 0.042, Kruskall-Wallis test). There were significantly more patients with seizures when the upper level weather type on the following day was WS then when it was NG, T, R, GNW, NES, or NWS type; fewer patients when the type was GNW (front side of the ridge) then when it was R or SWS; as well as when it was NES or GNW in comparison to SWS (Conover post-hoc analysis).

There is also a trend towards differences in upper level weather types on the following day when days are divided into day with or without patients with seizures (*p* = 0.060, χ^2^ test).

No differences were found when observing the surface synoptic situations (divided in four groups as discussed earlier) in relation to a daily number of patients with seizures (*p* = 0.481, Kruskall-Wallis test), as well as regarding the synoptic situations on a day (*p* = 0.316, Kruskall-Wallis test) or 2 days before (*p* = 0.305, Kruskall-Wallis test). Similarly to when observing the upper level weather types, differences seem to exist in synoptic situation types on the following day (*p* = 0.012, Kruskall-Wallis test), with fewer daily patients with seizures when the synoptic situation on the following day was high pressure field (*n* = 743) then when it was low pressure field (*n* = 279) or non-gradient pressure field (*n* = 457). These differences were maintained when observing just the colder part of the year (Nov-Apr, *p* = 0.038, Kruskall-Wallis test), but failed to reach significance in the warmer part of the year (May-Oct, Kruskall-Wallis test). Also noted was a difference (*p* = 0.039, χ^2^ test) in days with or without seizures depending on the synoptic situation on the following day; with days with no seizures being more prevalent when high pressure field was present on the subsequent day.

**Fig. 3:**
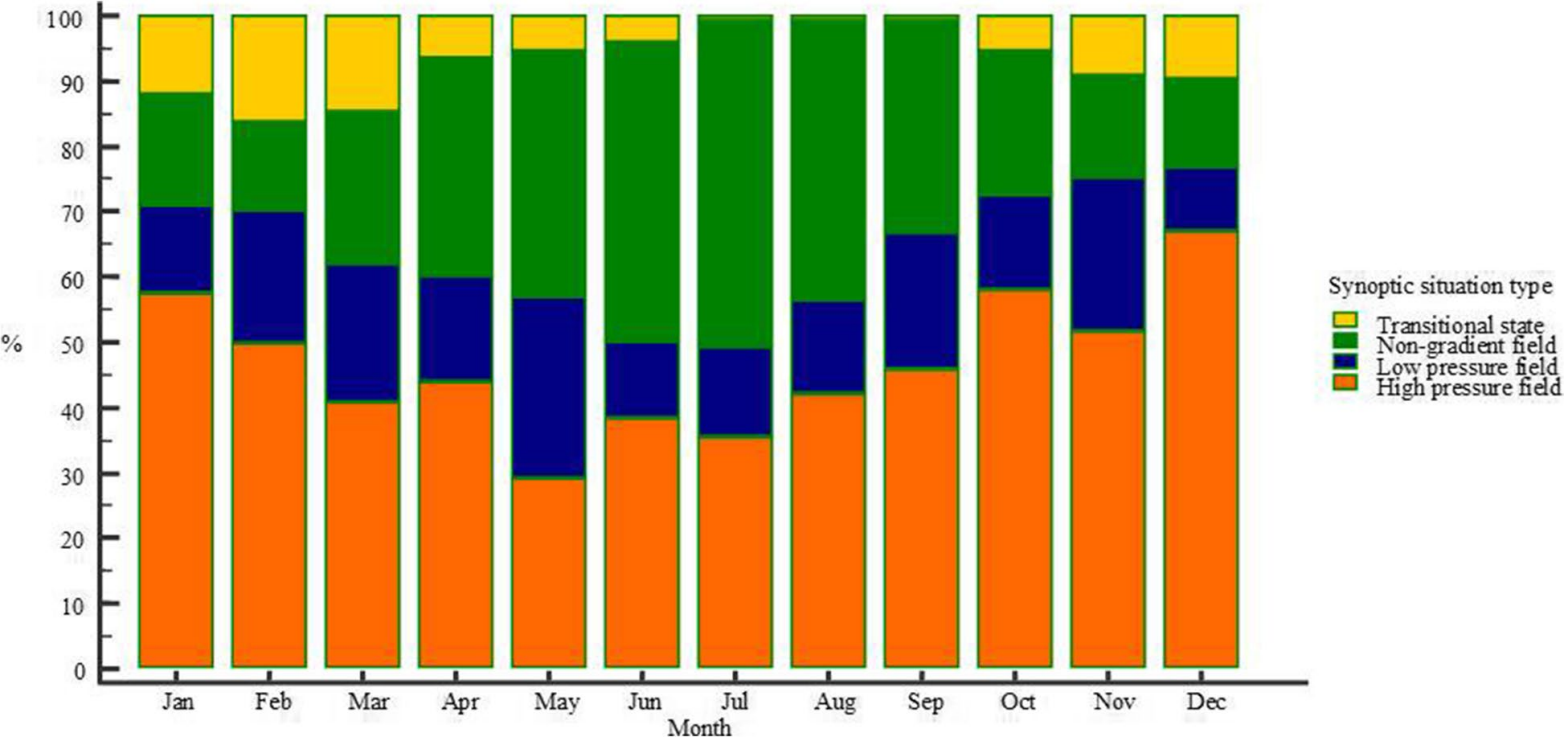
Monthly distribution of particular synoptic situation types, categorized into four basic groups

As in upper level weather types, synoptic situations were differently distributed on a monthly basis (*p* < 0.0001, χ^2^ test), as shown in Fig. 3. There were seasonal differences as well, as non-gradient field were more common in spring and summer, with fewer days in transitional states in summer. On the other hand, high pressure fields were more common in fall and winter (*p* < 0.0001, χ^2^ test).

Analyses of biometeorological situations provided further notable findings: when days were divided into favourable (*n* = 911) and unfavourable (*n* = 689) categories, there was again a difference according to this criterion on the following day in relation to the number of patients with seizures (*p* = 0.029, Mann-Whitney U-test), with fewer such patients if this prognosis was deemed favourable, a difference that was again maintained when days were divided into those with or without seizures (*p* = 0.044, χ^2^ test). On the same day, however, there were no differences according to this criterion (*p* = 0.699, Mann-Whitney U-test).

All of the aforementioned results are summarized in Table 2.

**Table 2:** Mean numbers of patients with seizures on particular days in vicinity of days with a particular upper level weather type, synoptic situation, or biometeorological forecast

	N	Seizure mean two days later	Seizure mean one day later	Seizure mean	Seizure mean one day before
Upper level weather types					
R	235	0.57 ± 0.73	0.57 ± 0.77	0.57 ± 0.77	0.63 ± 0.82
NG	121	0.58 ± 0.69	0.63 ± 0.82	0.59 ± 0.79	0.58 ± 0.76
GNW	286	0.63 ± 0.72	0.59 ± 0.76	0.59 ± 0.76	0.49 ± 0.74
NWS	121	0.62 ± 0.69	0.62 ± 0.66	0.62 ± 0.66	0.55 ± 0.63
GSW	104	0.61 ± 0.86	0.63 ± 0.80	0.63 ± 0.70	0.61 ± 0.76
SWS	287	0.64 ± 0.88	0.67 ± 0.89	0.68 ± 0.89	0.68 ± 0.79
T	96	0.67 ± 0.86	0.47 ± 0.82	0.47 ± 0.72	0.55 ± 0.86
ULC	108	0.54 ± 0.69	0.63 ± 0.75	0.63 ± 0.76	0.62 ± 0.82
NS	24	0.54 ± 0.66	0.46 ± 0.66	0.46 ± 0.66	0.63 ± 0.71
NES	17	0.53 ± 0.51	0.59 ± 0.51	0.59 ± 0.50	0.36 ± 0.79
SS	39	0.66 ± 0.71	0.49 ± 0.64	0.49 ± 0.64	0.67 ± 0.77
SES	18	0.33 ± 0.59	0.39 ± 0.69	0.39 ± 0.69	0.67 ± 1.03
WS	128	0.65 ± 0.82	0.69 ± 0.75	0.69 ± 0.75	0.80 ± 0.85
ES	15	0.60 ± 0.63	0.67 ± 0.81	0.67 ± 0.81	0.60 ± 0.83
*p*-value		0.982	0.577	0.720	**0.042**
Surface synoptic situations					
Low pressure field	279	0.68 ± 0.84	0.63 ± 0.83	0.62 ± 0.76	0.69 ± 0.84
High pressure field	743	0.58 ± 0.78	0.58 ± 0.79	0.58 ± 0.79	0.54 ± 0.74
Non-gradient pressure field	457	0.61 ± 0.76	0.69 ± 0.78	0.65 ± 0.79	0.64 ± 0.79
Transition state	121	0.59 ± 0.80	0.56 ± 0.69	0.60 ± 0.71	0.68 ± 0.82
*p*-value		0.305	0.316	0.501	**0.012**
Biometeorological forecast					
Favourable	911	0.63 ± 0.79	0.61 ± 0.79	0.59 ± 0.78	0.57 ± 0.78
Unfavourable	689	0.59 ± 0.78	0.61 ± 0.77	0.62 ± 0.79	0.65 ± 0.79
*p*-value		0.290	0.993	0.699	**0.029**

Values, arithmetic means±SD; *p*-values obtained using Kruskall-Wallis test when > 2 categories, Mann-Whitney U-test when 2 categories; *p* < 0.05 written in bold letters

We performed additional analyses using binary logistic regression with Wald χ^2^ test, wherein we used dichotomous quantifiers of seizure events on a given day. Days were thus sorted as those with or without patients with seizures. Additionally, we isolated days with two or more patients with seizures and performed similar analyses.

The results were once again most prominent regarding weather types on a following day: upper level weather types GNW and NES were less likely to happen on a day after a day with any seizure patients (*p* = 0.0037, OR 0.61 with 95% CI 0.44–0.85, and *p* = 0.042, OR 0.31 with 95% CI 0.09–0.96, respectively). On days after a day with at least two patients with seizures, GNW weather type was again less likely (*p* = 0.022, OR 0.54 with 95% CI 0.32–0.92). Instead of NES, in this case NWS weather type was less likely (*p* = 0.013, OR 0.35 with 95% CI 0.15–0.80). Contrary to earlier analyses, this test yielded a significant result regarding upper level weather types on a same day when any epileptic seizures occured: on those days, T upper level weather type was less likely (*p* = 0.017, OR 0.55 95% CI 0.35–0.90).

Regression analysis of surface weather types yielded results similar to those mentioned earlier. On a day following a day when there were any seizures, low pressure field and non-gradient weather types were more likely (*p* = 0.026, OR 1.36 with 95% CI 1.04–1.81, and *p* = 0.033, OR 1.28 with 95% CI 1.02–1.63, respectively). Similar results were seen regarding days with two or more seizure patients (low pressure field and non-gradient weather types more likely on a following day). However, on those days with two or more seizure patients, concurrent non-gradient weather type was more likely as well (*p* = 0.004, OR 1.66 with 95% CI 1.17–2.35).

Weather, whether upper level or surface, on previous days didn’t seem to have an influence on seizure events, similarly to earlier analyses.

**Fig. 4:**
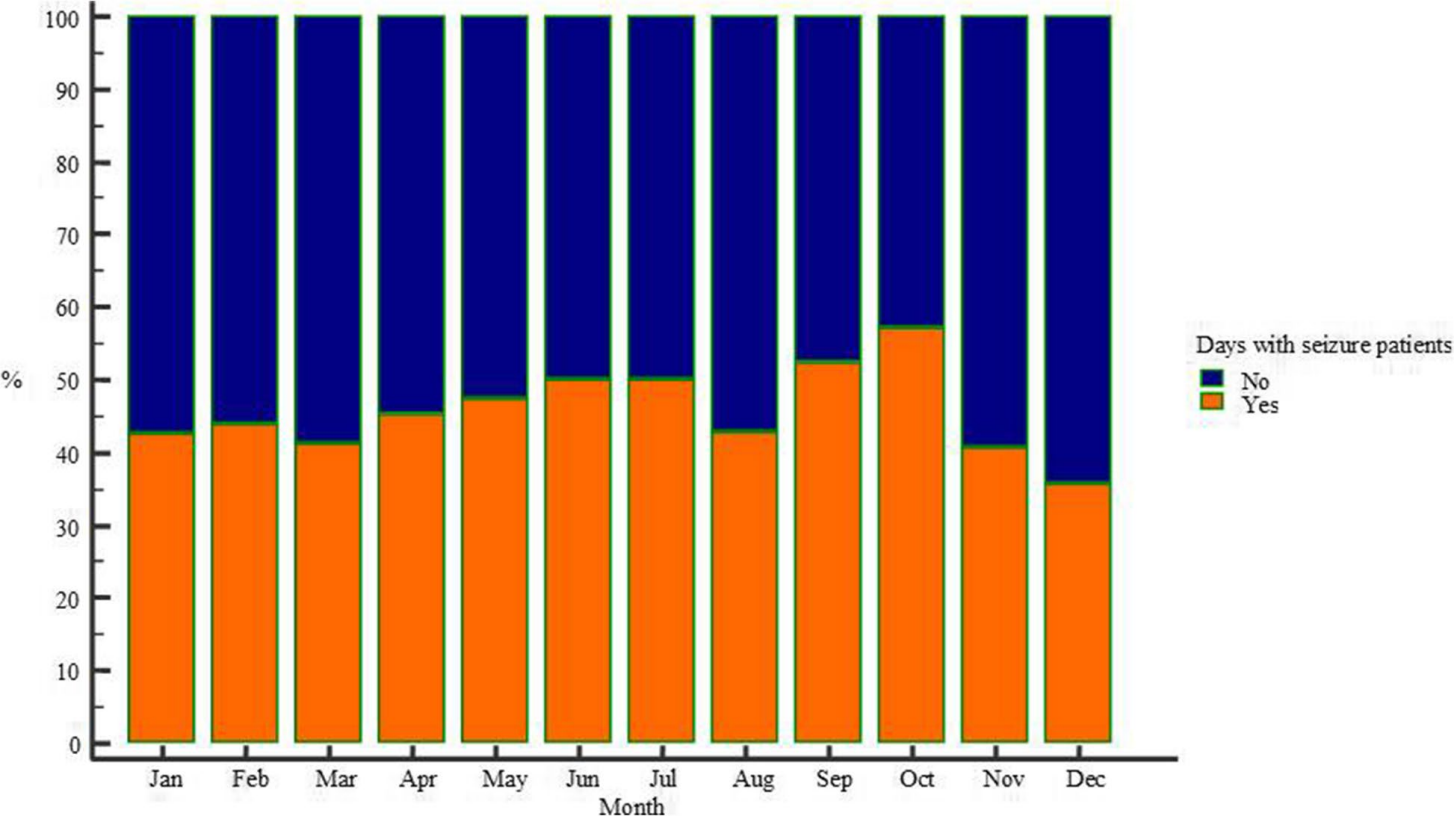
Monthly proportion of days with seizure patients

In addition to these aforementioned associations of seizures with short-term meteorological factors, there were differences in seasonal seizure occurrence as well. While monthly seizure distribution only showed a trend toward more patients with seizures in certain months (*p* = 0.063, Kruskall-Wallis test), as well as to differences in days with or without seizures in certain months (*p* = 0.053, χ^2^ test, Fig. 4), there was certainly a larger proportion of days with seizure patients in warmer (377/756, 49.87%), than in colder days of the year (353/844, 41.82%), *p* = 0.001, χ^2^ test.

Finally, it is important to note that a strong relationship exists between upper level weather types and surface synoptic situations (*p* < 0.0001, χ^2^ test), as well as between both of those factors and the biometeorological prognoses (*p* < 0.0001 for both, χ^2^ test).

## Discussion

Meteorological phenomena have long been thought of as modifying factors in various health-related conditions, but firm connections and solid scientific evidence is still scarce. They should therefore be studied in a more sophisticated way than just a linear correlation between one single meteorological parameter and a particular medical problem, as demonstrated in our results that point to a subtle metachronous interplay between various weather-related parameters and occurrence of epileptic seizures.

Weather types, both upper level and closer to the ground (surface weather types), don’t seem to influence the occurrence of epileptic seizures in the near future, or even in the time of their existence. On the other hand, all of the analyzed weather-related parameters seem to be associated with daily numbers of seizures on the previous day. The reason behind this phenomenon is uncertain, as there is no known mechanistic explanation that would enable patients with epilepsy to be influenced by future changes in the atmosphere. A part of this conundrum can potentially be explained by the fact that weather types are determined on 00 AM UTC, and are thus temporally much closer to at least a part of occurred seizures, which could have happened perhaps three or 4 hours before meteorological analyses were performed. This could mean that the analyses of the following day are in fact more precise for those patients than the analyses performed almost a day earlier, on 00 AM UTC that day (all seizures that occur after 12 AM on a given day are temporally closer to the weather assessments on the following day. Another possible explanation would be an unknown (probably environmental) factor that contributes to both imminent seizures and weather patterns in the near future (i.e. the next day).

A possible explanation for changes in seizure frequency could lie in atmospheric pressure changes. An earlier study [14] demonstrated that seizures were more common, with an odds ratio of 2.80, in known patients with epilepsy after a change in atmospheric pressure of 5.5 hPa during a single day. The validity of these observations is fortified by the fact that those seizures were recorded during video EEG monitoring, excluding the possibilities of other loss of consciousness (LOC) events. The rationale for seizure generation in these circumstances is not certain, but authors speculated that subtle changes of partial pressure of oxygen can lead to relative hyperventilation, a known factor in seizure generation, especially in the context of low partial oxygen pressure [37]. As atmospheric pressure changes certainly depend on weather types, either directly in the context of surface weather, or indirectly, via upper level steering effect, this provides at least partial explanation for our observations, especially as the type of upper level weather most associated with seizures was of a flow type, bound to result in pressure changes.

It would seem that all of the analyzed weather factors are related to a point, with 500 hPa pressure fields apparently confirming their “steering” effect on the synoptic situations below; while their strong associations with biometeorological prognoses demonstrate a consistency in their determination.

Occurrence of epileptic seizures seems to vary seasonally as well, as their frequencies seem to peak in summer months, with a sharp drop from September and October to November and December. These results are contrary to results of other studies that showed higher seizure frequency during winter and autumn months and days with lower daylight duration [18, 24]. A rationale for a higher prevalence of epileptic seizures on warmer day could be down to certain physiological processes. One might envision that dehydration, loss of sodium through sweating, or altered pharmacokinetics of antiepileptic drugs could have a deleterious effect on seizure generation. Our results could also partially stem from a heterogenous distribution of upper level weather types and synoptic situations over the year.

The limitations of our study include the fact that we can’t exclude the presence of confounders related to weather, as the connection between them and human health (and seizure disorders in particular) is not yet fully understood. Secondly, we can’t estimate the proportion of patients that were admitted as an epileptic seizure, but in reality had a psychogenic non-epileptic seizure (PNES). This is particularly important; as weather is known to influence psychiatric disorders as well [38]. Future studies could probably be improved by monitoring seizure events in a specialized setting, as an epileptology specialist is more educated to differentiate between true epileptic seizures, PNES, or other LOC events. Another useful variable to include in analyses would be the time of day when a seizure occurred, as it would enable the researchers to pinpoint actual coexistent weather conditions more precisely.

## Conclusions

Although these findings deducted from long term weather analyses and a large sample of patients reveal certain trends, it would not seem reasonable to appoint an unordinate amount of importance to the effect the weather exerts on patients with epilepsy, as the effect size is likely relatively small. However, it would be reasonable to conclude that weather patterns in fact have a certain influence on patients with epilepsy, and possibly on overall human health. Therefore, further studies should be encouraged to elucidate the mechanisms in which weather conditions, particularly pressure patterns, i.e. synoptic situation, can influence episodic, abrupt and paroxysmal disorders and symptoms in neurology, as well as in other fields of medicine.

## Data Availability

Data used for analyses is available at: https://figshare.com/articles/dataset/Meteorology_epilepsy/14185142

## Abbreviations

EEG: Electroencephalography
ECMWF: European Centre for Medium- Range Weather Forecast
LOC: Loss of consciousness
PNES: Psychological non-epileptic seizure
UTC: Coordinated Universal Time
R: Ridge
NG: Non-gradient field
NW: North-westerly flow
SW: South-westerly flow
W: Westerly flow
T: Trough
ULC: Upper level low–cyclone
DWD: Deutsche Wetterdienst.
GNW: Front side of the ridge
NES: Northeasterly flow
NWS: Back side of the trough
GNW: Front side of the ridge
SWS: Front side of the trough
